# Prosthetic joint infection caused by *Neisseria sicca*/*subflava*

**DOI:** 10.1186/s13256-026-05838-x

**Published:** 2026-02-02

**Authors:** Hassaan Abid, Akrum Saleh, Michael DeBrota, Hamza Waheed, Rodney Yuhico, Ritika Zijoo

**Affiliations:** Indiana University School of Medicine (IUSM), Indianapolis, USA

**Keywords:** Prosthetic joint infection, *Neisseria sicca*/*subflava*, Late-onset infection, Case report

## Abstract

**Background:**

Prosthetic joint infections are most commonly caused by staphylococci and streptococci, while commensal *Neisseria* species are exceedingly rare etiologic agents.

**Case presentation:**

We report a 71-year-old white male with multiple comorbidities who presented with progressive left knee pain 9 years after total knee arthroplasty. Synovial fluid analysis showed an elevated leukocyte count with neutrophil predominance, and cultures subsequently grew *Neisseria sicca*/*subflava*. Given the concern for prosthetic joint infection, he underwent prosthesis explantation and received intravenous ceftriaxone 2 g daily for 6 weeks. Poor dentition was identified as a suspected potential source of hematogenous seeding. The patient demonstrated clinical improvement with surgical intervention and targeted antimicrobial therapy.

**Conclusion:**

*Neisseria sicca*/*subflava* should be recognized as a rare but clinically significant cause of prosthetic joint infection.

## Introduction

Prosthetic joint infections (PJIs) occur in the tissues surrounding a joint prosthesis, such as hip, knee, or shoulder implants, and differ significantly from native joint infections owing to the ability of microorganisms to adhere to prosthetic material and form biofilms. According to a review in *The New England Journal of Medicine*, the most common organisms causing PJI are coagulase-negative staphylococci (37%), followed by *Staphylococcus aureus* (24%), *Streptococcus* species (14%), *Enterococcus* species (8%), *Cutibacterium* species (8%), and Enterobacterales (7%). Less common organisms include *Corynebacterium* species, *Pseudomonas* species, fungi, and mycobacteria [1].

Commensal *Neisseria* species are infrequently associated with musculoskeletal infections. Existing reports describe isolated cases of vertebral osteomyelitis, discitis, and endocarditis, but joint involvement remains exceedingly rare [2–6]. A recent literature review identified only a small number of osteomyelitis cases attributed to *N. sicca*, and current IDSA guidelines do not include it among pathogens implicated in septic arthritis [3, 4]. Given the scarcity of reported cases, further characterization of *N. sicca* pathogenicity is warranted.

## Case description

A 71-year-old white male presented with worsening left knee pain. His past medical history was significant for morbid obesity, coronary artery disease, heart failure with reduced ejection fraction, paroxysmal atrial fibrillation, hypertension, and hyperlipidemia. Past surgical history included right total hip arthroplasty (2017), left total knee arthroplasty (2015), and placement of an implantable cardioverter-defibrillator.

He reported a sudden onset of localized, nonradiating pain while rising from a chair, progressively worsening over 2 months. He denied trauma, falls, fever, or chills. Examination revealed swelling, warmth, erythema, and mild pain with external rotation.

A knee aspiration drained 20 cc of straw-colored, blood-tinged fluid. The initial assessment favored arthritis, and he received acetaminophen while awaiting results. The results for the synovial fluid analysis are presented in Table 1.

**Table 1:** Synovial Fluid Analysis

Test	Result
Color	Red
Clarity	Turbid
Total nucleated cells	7237/µL
White blood cell (WBC) count	7220/µL
Red blood cell (RBC) count	21,000/µL
Polymorphonuclear neutrophils (PMNs)	80%
Crystals	None
Gram stain	Many WBCs; no organisms
Final culture	*Neisseria sicca*/*subflava*
Susceptibility	Not performed

Additional inflammatory markers showed ESR 98 mm/hr and CRP 4.2 mg/dL.

The diagnostic evaluation was guided by the 2018 Musculoskeletal Infection Society (MSIS)/International Consensus Meeting (ICM) criteria for prosthetic joint infection, which integrate clinical findings, inflammatory markers, synovial fluid analysis, and microbiologic data. Although the synovial leukocyte count did not meet major MSIS/ICM criteria for acute prosthetic joint infection, chronic and low-grade PJIs, particularly those caused by low-virulence organisms, may present with lower synovial leukocyte counts and attenuated inflammatory responses, especially in late-onset infections occurring years after implantation. When interpreted alongside markedly elevated inflammatory markers, progressive clinical symptoms, and a positive synovial fluid culture, the overall clinical picture was felt to be most consistent with a probable chronic prosthetic joint infection rather than a noninfectious arthropathy.

Synovial fluid cultures were obtained via sterile joint aspiration at an outside facility before transfer. Detailed information regarding specific culture media (aerobic, anaerobic, or enrichment) and incubation conditions was not available. No antibiotics were administered prior to joint aspiration. The organism was reported as *Neisseria sicca*/*subflava* using routine clinical laboratory identification methods at the referring institution. Blood cultures obtained before antibiotic initiation at our institution remained negative. Repeat intraoperative cultures obtained at the time of prosthesis explantation were also negative.

Given the positive synovial culture and clinical concern for prosthetic joint infection, the patient was transferred for urgent prosthesis explantation and initiated on intravenous ceftriaxone 2 g daily. The infectious diseases team noted poor dentition as a suspected potential source of hematogenous seeding, given the organism’s known oropharyngeal colonization. He recovered uneventfully with plans for 6 weeks of intravenous antimicrobial therapy.

At the time of manuscript submission, the patient continued treatment with ongoing symptomatic and functional improvement.

## Discussion

This case highlights an exceptionally rare cause of prosthetic joint infection. While most PJIs involve staphylococci, streptococci, or enteric Gram-negative bacilli, *Neisseria sicca*/*subflava* has not been well described as a causative pathogen in prosthetic joint infection. A focused literature search was conducted using PubMed and Google Scholar with combinations of the terms “Neisseria sicca,” “Neisseria subflava,” “prosthetic joint infection,” and “periprosthetic joint infection.” While prior reports describe invasive infections such as osteomyelitis, discitis, and endocarditis caused by *N. sicca*, no published cases of prosthetic joint infection attributable to *Neisseria sicca*/*subflava* were identified in the available English-language literature [3–7].

Commensal *Neisseria* species are often dismissed as contaminants; however, several features supported the interpretation of this isolate as a true pathogen rather than contamination. These included sterile sampling technique, absence of prior antibiotic exposure, elevated inflammatory markers, progressive clinical symptoms, and subsequent clinical improvement following prosthesis explantation and targeted antimicrobial therapy.

The presence of a positive preoperative synovial fluid culture with negative intraoperative explant cultures introduces diagnostic complexity. Potential explanations include low bacterial burden, fastidious organism growth characteristics, and sampling variability. Discordant culture results are well described in chronic, low-grade prosthetic joint infections and do not exclude infection when clinical, laboratory, and microbiologic features collectively support the diagnosis.

Treatment strategies for such rare pathogens are not well defined, as existing guidelines do not address commensal *Neisseria* species [8, 9]. However, most *Neisseria* species are susceptible to third-generation cephalosporins, supporting our approach of 6 weeks of ceftriaxone following prosthesis removal.

Our case report fills several important gaps in medical literature:

1. Pathogenic expansion: it demonstrates that *N. sicca*/*subflava*, typically regarded as a benign oral commensal, can cause late-onset PJI.
2. Therapeutic uncertainty: it underscores the absence of susceptibility data or guideline-based therapy for this organism.
3. Microbiome consideration: it emphasizes the need to evaluate oral health as a potential source of atypical PJIs.

## Conclusion

This case demonstrates that even low-virulence commensal organisms such as *Neisseria sicca*/*subflava* can cause prosthetic joint infection. It underscores the importance of applying established diagnostic frameworks, such as MSIS/ICM criteria, while incorporating clinical judgment in atypical presentations. Consideration of uncommon pathogens is particularly important in late-onset prosthetic joint infections, especially in patients with potential risk factors such as poor oral health.

## Data Availability

Not applicable.

## Abbreviations

PJI: Prosthetic joint infection
PJIs: Prosthetic joint infections
WBC: White blood cell
RBC: Red blood cell
PMNs: Polymorphonuclear neutrophils
ESR: Erythrocyte sedimentation rate
CRP: C-reactive protein
MSIS: Musculoskeletal Infection Society
ICM: International consensus meeting
IDSA: Infectious Diseases Society of America
IV: Intravenous
ICD: Implantable cardioverter-defibrillator
*N.*: *Neisseria* (genus abbreviation, for example, *N. sicca*)

## References

[1] Patel R. Periprosthetic joint infection. N Engl J Med. 2023;388:251–62. 36652356 10.1056/NEJMra2203477

[2] Neisseria—An overview. ScienceDirect Topics.

[3] Dyal NP, Orenstein R, Nagarakanti SR. *Neisseria sicca* vertebral osteomyelitis. J Clin Med. 2024;13: 7241. 39685699 10.3390/jcm13237241 PMC11642248

[4] Tamma PD, et al. IDSA guidance on resistant gram-negative infections. *Clin Infect Dis.* 2023. 10.1093/cid/ciad428 37463564

[5] Guide to utilization of the microbiology laboratory. IDSA/ASM. 2024.

[6] Heiddal S, et al. *Neisseria sicca* native-valve endocarditis. Clin Infect Dis. 1993. 10.1093/clind/16.5.667 8507758

[7] Browning S, et al. *Neisseria meningitidis* PJIs. J Bone Jt Infect. 2020. 10.5194/jbji-6-33-2020 PMC7517647 32983846

[8] UC Davis Health. Overview of PJI diagnosis. 2021.

[9] Kapadia BH, et al. Periprosthetic joint infection. Lancet. 2016. 10.1016/S0140-6736(14)61798-0. 26135702 10.1016/S0140-6736(14)61798-0

